# *Bifidobacterium* Species Colonization in Infancy: A Global Cross-Sectional Comparison by Population History of Breastfeeding

**DOI:** 10.3390/nu14071423

**Published:** 2022-03-29

**Authors:** Diana H. Taft, Zachery T. Lewis, Nhu Nguyen, Steve Ho, Chad Masarweh, Vanessa Dunne-Castagna, Daniel J. Tancredi, M. Nazmul Huda, Charles B. Stephensen, Katie Hinde, Erika von Mutius, Pirkka V. Kirjavainen, Jean-Charles Dalphin, Roger Lauener, Josef Riedler, Jennifer T. Smilowitz, J. Bruce German, Ardythe L. Morrow, David A. Mills

**Affiliations:** 1 Department of Food Science and Technology, University of California Davis, Davis, CA 95616, USA; dhtaft@ucdavis.edu (D.H.T.); ztlewis@ucdavis.edu (Z.T.L.); nhqnguyen@ucdavis.edu (N.N.); stvho@ucdavis.edu (S.H.); cfmasarweh@ucdavis.edu (C.M.); vpdunnecastagna@ucdavis.edu (V.D.-C.); jensm@ucdavis.edu (J.T.S.); jbgerman@ucdavis.edu (J.B.G.); 2 Foods for Health Institute, University of California Davis, Davis, CA 95616, USA; 3 Department of Pediatrics, University of California Davis School of Medicine, Sacramento, CA 95817, USA; djtancredi@ucdavis.edu; 4 US Department of Agriculture, Western Human Nutrition Research Center, Davis, CA 95616, USA; mnhuda@ucdavis.edu (M.N.H.); charles.stephensen@ars.usda.gov (C.B.S.); 5 Department of Nutrition, University of California Davis, Davis, CA 95616, USA; 6 Center for Evolution and Medicine, School of Human Evolution and Social Change, Arizona State University, Tempe, AZ 85281, USA; katie.hinde@asu.edu; 7 Dr. von Hauner Children’s Hospital, Ludwig Maximilian University, 80337 Munich, Germany; erika.von.mutius@med.uni-muenchen.de; 8 Institute for Asthma and Allergy Prevention, Helmholtz Centre Munich, 85764 Neuherberg, Germany; 9 Environment Health Unit, National Institute for Health and Welfare, 70210 Kuopio, Finland; pirkka.kirjavainen@thl.fi; 10 Institute of Public Health and Clinical Nutrition, University of Eastern Finland, 70211 Kuopio, Finland; 11 Department of Respiratory Disease, UMR/CNRS 6249 Chrono-Environment, University Hospital of Besançon, F-25000 Besançon, France; charles.dalphin@univ-fcomte.fr; 12 Christine Kühne-Center for Allergy Research and Education, 7265 Davos, Switzerland; roger.lauener@kispisg.ch; 13 Children’s Hospital of Eastern Switzerland, 9000 St. Gallen, Switzerland; 14 Children’s Hospital Schwarzach, 5620 Schwarzach, Austria; josef.riedler@kh-schwarzach.at; 15 Department of Environmental and Public Health Sciences, University of Cincinnati, Cincinnati, OH 45221, USA; 16 Department of Viticulture and Enology, University of California Davis, Davis, CA 95616, USA

**Keywords:** breastfeeding, *Bifidobacterium*, microbial extinction, infants

## Abstract

*Bifidobacterium* species are beneficial and dominant members of the breastfed infant gut microbiome; however, their health benefits are partially species-dependent. Here, we characterize the species and subspecies of *Bifidobacterium* in breastfed infants around the world to consider the potential impact of a historic dietary shift on the disappearance of *B. longum* subsp. *infantis* in some populations. Across populations, three distinct patterns of *Bifidobacterium* colonization emerged: (1) The dominance of *Bifidobacterium longum* subspecies *infantis*, (2) prevalent *Bifidobacterium* of multiple species, and (3) the frequent absence of any *Bifidobacterium.* These patterns appear related to a country’s history of breastfeeding, with infants in countries with historically high rates of long-duration breastfeeding more likely to be colonized by *B. longum* subspecies *infantis* compared with infants in countries with histories of shorter-duration breastfeeding. In addition, the timing of infant colonization with *B. longum* subsp. *infantis* is consistent with horizontal transmission of this subspecies, rather than the vertical transmission previously reported for other *Bifidobacterium* species. These findings highlight the need to consider historical and cultural influences on the prevalence of gut commensals and the need to understand epidemiological transmission patterns of *Bifidobacterium* and other major commensals.

## 1. Introduction

*Bifidobacterium* colonization has a number of potential health benefits for infants, including a reduction of allergies [1,2], improved vaccine response [3], reduced carriage of antimicrobial resistance genes [4], reduced carriage of virulence factor genes [5], reduced enteric inflammation [6], and immunoregulation via microbial metabolites [7]. However, some of these health benefits are dependent on which species of *Bifidobacterium* are present in the gut microbiome [3,8].

Despite the importance of *Bifidobacterium* for infant health, there is evidence that at least one subspecies, *Bifidobacterium longum* subsp. *infantis,* is in danger of extinction [9,10]. The extinction of gut commensals is a growing concern, because of the potential for health and immunological disruptions associated with the loss of commensal microbes [11]. A number of factors contribute to the loss of microbial diversity, including disruptions to vertical transmission (for example, via C-section and intrapartum antibiotic use) and disruptions to horizontal transmission (notably, via dietary shifts) [12].

Changes to diet alone can drive extinctions in the gut microbiome over generations [13]. Such extinctions occur by removing microbial-accessible carbohydrates (MAC) from the diet [13]. MACs are carbohydrates that are available for fermentation by the microbiome but are not digested by the host. Loss of MACs from the mouse diet drive irreversible losses of commensal species as the microbes that would otherwise consume those MACs decline, and so are unable to return to populations even in subsequent generations again fed a high-MAC diet [13]. 

The concept of MAC-driven extinctions is potentially relevant to understanding the modern, breast-fed infant gut microbiome. Diet is a major driver of the infant gut microbiome [14]. The earliest studies of the infant gut microbiome were conducted a century ago and highlighted the difference between breastfed infants and formula-fed infants, with breastfed infants exhibiting near monocultures of *Bifidobacterium* and formula-fed infants exhibiting a more mixed microbiome without *Bifidobacterium* [15]. The ability of *Bifidobacterium* to dominate the breast-fed infant gut is driven, in part, by human milk oligosaccharides (HMOs) [16,17,18], the third most abundant solid component of human milk [19]. Despite the abundance of HMOs in human milk, these specialized carbohydrates are not digested by the infant [20]. Instead, HMOs act as MACs for beneficial bacteria, including *Bifidobacterium*, and as decoy receptors for pathogens [21]. Some *Bifidobacterium* species are efficient consumers of HMOs, to the point that HMOs are identified as the “bifidogenic factor” of human milk [16,22]. As a result, a breastfed infant exposed to an HMO-consuming species of *Bifidobacterium* from the mother or from other environmental sources will likely have very high levels of that species of *Bifidobacterium*.

Human milk substitutes have typically lacked HMOs, although a few formulas first introduced in 2018 do contain a single HMO. Despite this, HMOs are not typically included in infant formula, and when they are included, the diversity of HMO structures is far below that found in human milk. As a result, infants fed artificial formulas consume a diet low in HMOs, and therefore a diet that is low MAC.

Not all *Bifidobacterium* are equally efficient at consuming HMOs [23]. *Bifidobacterium* is a genus composed of 54 different species that had been identified as of January 2017 [24]. Some *Bifidobacterium,* such as *B. longum* subsp. *infantis,* are efficient consumers of HMOs but lack the ability to consume plant oligosaccharides, while others, such as *B. longum* subsp. *longum,* are better adapted for the consumption of plant oligosaccharides but some strains have the ability to also consume a subset of HMOs [23]. This creates the possibility that while some species of *Bifidobacterium* are likely to be members of the adult microbiome and to undergo vertical transmission from the mother [25], others such as *B. longum* subsp. *infantis* may rely on horizontal transmission between breastfed infants for ongoing persistence in populations. These species may be uniquely at risk of extinction should a period of disruption to breastfeeding occur in a population, as enough breastfed infants must come into contact with other breastfed infants already colonized by an HMO-dependent *Bifidobacterium* to maintain sustained transmission of the species. In fact, there is already some evidence that this extinction has occurred in Western populations as *B. longum* subsp. *infantis* has become increasingly difficult to detect in these populations, even in breastfed infants never exposed to antibiotics [10]. Therefore, historical breastfeeding patterns must be considered in addition to current breastfeeding in order to understand the relationship between breastfeeding and the infant gut microbiome in the present day. Infants from populations that experienced periods of lower breastfeeding initiation and shorter durations may be living in regions where HMO-consuming *Bifidobacterium* are near extinction due to the loss in prior generations. Should *B. longum* subsp. *infantis* be at risk of extinction due to low breastfeeding rates, we would expect to find all of the following to be true: (1) It would be found at lower rates in populations with historically lower breastfeeding rates, even if breastfeeding rates have rebounded in the present day; (2) in populations with lower breastfeeding rates, we would expect delayed colonization by *B. longum* subsp. *infantis* compared to other species of *Bifidobacterium* because it will take longer for infants to come into contact with the bacterium; and (3) we would expect that *B. longum* subsp. *infantis* would be lost from the gut microbiome as breastfeeding ends due to the loss of HMO access. Conversely, if the presence of *B. longum* subsp. *infantis* is not tightly tied to HMO consumption, it will remain in the adult population and will not be at risk of extinction due to infant diet changes. In brief, we hypothesize that commensals such as *B. longum* subsp. *infantis* dependent on HMO consumption may be lost from populations with a history of lower breastfeeding initiation and duration while *Bifidobacterium* species capable of consuming both HMOs and plant oligosaccharides may have a better chance at surviving in populations that have experienced low breastfeeding rates. Here we work to describe the species of *Bifidobacterium* found in global infant populations and consider how the population history of breastfeeding practices and longitudinal observations on infant *Bifidobacterium* colonization relate to the currently observed patterns.

## 2. Methods

### 2.1. Inclusion Criteria for Cohorts

For this analysis, published and unpublished cohorts of term infants where at least some infants were breastfed were selected for inclusion. Cohorts needed to have a minimum of 20 infants and have species-level data on the relative abundance of the *Bifidobacterium* as measured by 16S rRNA gene sequencing and *Bifidobacterium*-specific terminal restriction fragment length polymorphism (Bif-TRFLP) and *Bifidobacterium* Longum-Infantis Ratio (BLIR) analyses in infant stools between the ages of 1 or 2 months or have 16S rRNA gene sequencing data available with DNA available for species-level analysis of *Bifidobacterium*. Publicly available datasets were included from Gambia [26] and Bangladesh [3]. The PASTURE cohorts from Austria, Finland, Germany, and Switzerland are previously published [27,28], although this is the first publication to include species-level *Bifidobacterium* data from the PASTURE cohorts. Additionally, data from a subset of infants enrolled in the University of California Davis Lactation Study (Davis, CA, USA) [29,30] and a subset of infants enrolled in the Pediatric Respiratory and Enteric Virus Acquisition and Immunogenesis Longitudinal (PREVAIL) study (Cincinnati, OH, USA) [31] were included. Table 1 summarizes the cohorts and lists the total number of infants in each cohort, the number of infants who had a sample from the ages of 1 to 2 months for inclusion in this study, and the number of infants who were at least partially breastfed at the time of sample collection. Historical breastfeeding patterns were defined by searching the literature for references on breastfeeding rates published between 1900 and the present day. A high breastfeeding pattern was defined as one where breastfeeding initiation was nearly universal and breastfeeding duration was typically longer than 1 year. The medium breastfeeding pattern was defined as high current and past rates of breastfeeding initiation, but with evidence of historically short (median less than 6 months) duration. The low breastfeeding pattern was defined as occurring in the case of a documented period where at least half of infants were never breastfed (meaning breastfeeding was not initiated), regardless of the current population’s breastfeeding initiation and duration. The medium and low historical breastfeeding patterns are distinct because most infants in the medium breastfeeding pattern were consistently breastfed for at least brief periods of time, while there was a measurable and recorded period when the majority of infants in low historical breastfeeding pattern regions never received even a single feeding of breastmilk. While breastfeeding rates for each cohort are calculated using all infants with available breastfeeding data in each cohort (Table 1), only infants who were at least partially breastfed at the time of the sample taken at month 1 or 2 are included in all subsequent analyses in this paper. The UC Davis Lactation Study was approved by the UC Davis Institutional Review Board (Protocol ID 216198, first approved 22 February 2011), and all mothers provided written informed consent for their and their infant’s participation in this study. The PREVAIL cohort was approved by the Centers for Disease Control and Prevention (CDC) Institutional Review Board (Number 6952) and the Cincinnati Children’s Hospital Medical Center Institutional Review Board (Protocol Number 2016_9093, 21 February 2017), and all mothers provided written informed consent for their and their infant’s participation in this study. For the PASTURE cohorts, the study was approved by local research ethics committees, and written informed consent was obtained from the infant’s parents. The Bangladeshi cohort was approved by the Research Review Committee (RRC) and Ethical Review Committee (ERC) of the International Centre for Diarrhoeal Disease Research, Bangladesh (icddr,b, Protocol #PR—13068, approved 2 December 2013). UC Davis IRB also approved the protocol (549272-6, 21 February 2014). Mothers provided written informed consent. For the Gambian cohort, all data used for this project were publicly available [26].

For each cohort, data on the median duration and initiation rates of breastfeeding are reported if available for the cohort. In addition, historical breastfeeding practices of each country of origin of a cohort are described based on reviews of the published literature. In addition to the month 1–2 samples described above, six cohorts had longitudinal samples available. The Bangladesh cohort had additional samples from a subset of infants aged 2 years with 16S rRNA gene sequencing and Bif-TRFLP/BLIR. The four PASTURE cohorts had additional samples from the age of 1 year on a subset of infants. The Davis cohort had additional samples from a subset of 3-day-old and 1-month-old infants with 16S rRNA gene sequencing and Bif-TRFLP/BLIR analyses. These cohorts will permit a limited examination of *Bifidobacterium* species colonization over time.

### 2.2. 16S rRNA Gene Sequencing and Bif-TRFLP/BLIR

All infant stool samples were extracted and sequenced as described in Davis et al. [23] Analysis of the 16S rRNA gene sequencing results of all raw data files were completed using QIIME2 [32] (version qiime2-2017.8) and DADA2 [33]. The identification of *Bifidobacterium* species and *B. longum* subspecies were completed using *Bifidobacterium*-specific terminal restriction fragment length polymorphism (Bif-TRFLP) and the *Bifidobacterium* Longum-Infantis Ratio (BLIR) as described in Davis et al. [26,34] Bif-TRFLP and BLIR are well validated by culture for the identification of *Bifidobacterium* species and subspecies [26,34], and enable efficient and cost-effective identification of *Bifidobacterium* species in a large number of samples.

The relative abundance of total *Bifidobacterium* was compared by cohort and historical breastfeeding pattern using a Kruskal–Wallis test, followed by Dunn’s test with Bonferroni correction if the results were significant. The prevalence of *Bifidobacterium* species present in at least two cohorts was compared based on breastfeeding history in each cohort using generalized estimating equations (GEE) as implemented in the gee package in R version 3.6.3 [35] with a binomial family and logit linker, the cohort of origin was used as the clustering variable, and using an exchangeable correlation structure. A *Bifidobacterium* species was considered present in an infant if there was any detectable level of that species in the infant’s microbiome. Infants who receive a smaller portion of breastmilk in their diet are likely to have lower levels of *Bifidobacterium*, but any breastmilk in the diet will ensure that infants are consuming at least some amount of HMOs. By focusing on any breastfeeding and the presence or absence of *Bifidobacterium* species in infants fed at least some breastmilk, this analysis is focused on the prevalence of *Bifidobacterium* in populations where an infant exposed to a *Bifidobacterium* has a reasonable chance of having any detectable level of that *Bifidobacterium.* A *Bifidobacterium* species was considered present in a cohort if that species was present in at least one infant, as any detection of that species indicates it has not been eradicated. The species included for this analysis were *B. adolescentis, B. animalis, B. bifidum, B. breve, B. longum* subsp. *infantis, B. longum* subsp. *longum, B. longum* of unknown subsp., and *B. pseudocatenulatum.* This meant eight separate models were constructed, leading to a Bonferroni corrected *p*-value of less than 0.0062 to be considered significant after adjusting for multiple comparisons. 

To understand the extent to which individual species of *Bifidobacterium* shape the microbiome, we sought to identify species that could dominate (>50% relative abundance) the infant gut microbiome. To do this, for each species of *Bifidobacterium* in each cohort, we sought to identify infants whose microbiome had >50% relative abundance of a single species or subspecies of *Bifidobacterium*.

Finally, in the cohorts with samples from infants collected at older ages, Chi-square tests were used to compare the chances of detecting *B. longum* subsp. *infantis* in infants who were still breastfed compared to those who were weaned.

## 3. Results

### 3.1. Study Cohorts and Breastfeeding Patterns

This study included eight cohorts with a total of 979 breastfed infants with stool samples collected at age 1 or 2 months. Two cohorts were conducted in two low-income countries—Bangladesh and the Gambia—and six cohorts were conducted in five high-income countries—Austria, Finland, Germany, Switzerland, and the United States (US). The countries included have three historically different breastfeeding patterns, described here as “high”, “medium”, and “low” breastfeeding.

The first breastfeeding pattern, classified as “high”, is characterized by consistent, high rates of breastfeeding initiation and long durations of breastfeeding without evidence of historical interruptions in either breastfeeding initiation or duration. This is the pattern observed in the Gambia and Bangladesh. In the 1980s, when breastfeeding was low in much of the world, 97.5% of Bangladeshi children were fed at least some breastmilk and the mean duration of breastfeeding was 26 months [36]. Furthermore, this pattern of high rates of breastfeeding and long duration remained consistent in studies of Bangladesh from the 1970s through to the 1990s [37]. More recently, the initiation of breastfeeding in Bangladesh remains high (98.3%) and the duration of breastfeeding has increased to a mean of 31.9 months [38]. The Gambia has a similar historical and modern pattern, where a report from 1979 points out that “all Gambian women breast feed their babies for up to two years” [39]. Despite a shorter-than-optimal duration of exclusive breastfeeding, breastfeeding rates remain high in the Gambia today, with a median duration of 20 months with over 95% of infants ever breastfed [40]. Therefore, in this study, infants in the Bangladesh and Gambian cohorts were therefore considered to have a “high” historical breastfeeding pattern. The median duration of breastfeeding is unknown for the Gambian cohort, but all infants in this group were breastfed for at least 5 months as inclusion criteria included the availability of week 20 milk samples [26]. The median duration of breastfeeding in the Bangladeshi cohort is also unknown as infants in this cohort were only followed until age 2 years, and more than half of the infants enrolled in the study were still breastfed at this time point (Table 2). This means that the high breastfeeding pattern is still observed in the present day in these countries.

The second breastfeeding pattern, classified as “medium”, is characterized by high, consistent breastfeeding initiation but historical evidence of a short duration of breastfeeding (less than 6 months of any breastfeeding in the majority of infants). This pattern is observed in Austria, Finland, Germany, and Switzerland, with historical documentation of a median duration of breastfeeding of less than 6 months during the 1970s and 1980s. In European countries, there was a general decline in breastfeeding following World War II followed by increased rates of breastfeeding starting in the 1970s [41]. In Austria, Germany, and Finland, in the 1970s and 1980s, most women initiated breastfeeding, but the duration was short [42]. In Austria, only 5% of infants were breastfed at 3 months post-partum in 1980, which increased to 41% by 1984 [43], indicating that the majority of infants did not receive a full six months of breastfeeding during the early 1980s. In Finland in the early 1980s, most women initiated breastfeeding, but only one-third of infants were breastfed until 3 months [44], again indicating a historical period where the majority of infants were not at least partially breastfed for six months. In Germany, only 2% of infants were still breastfed at the age of 6 months in 1980 [45]. In Switzerland, in 1978, 92% of mothers initiated breastfeeding, but only 30% were still breastfeeding by 4 months [46]. These past patterns are no longer present in these countries. In Austria today, breastfeeding initiation rates are currently approximately 98% with a median duration of 27 weeks [47]. In Finland today, almost all mothers initiate breastfeeding, 60% are still breastfeeding at 6 months, and one-third are still breastfed at 11 months of age [48]. In Germany, 90% of women initiate breastfeeding and more than half of all infants are still breastfed at 6 months [49]. In Switzerland, breastfeeding initiation is 95% and the median duration of breastfeeding is 31 weeks [50]. These numbers are consistent with those observed for the study cohorts (Table 2). Therefore, even though present-day practices in these countries would qualify for the “high” category, historical evidence supports a period with low HMO consumption by infants creating the opportunity for shifts in infant *Bifidobacterium* populations.

The third pattern of breastfeeding, classified as “low”, is characterized by low historic rates of breastfeeding initiation. The two cohorts from the United States meet this pattern, as the United States experienced a low point in breastfeeding in 1971 when less than 1 in 4 women breastfed their infants even once [51]. Following sustained public health efforts, rates partially recovered to approximately 50–60% initiating breastfeeding in the 1980s [52]. Breastfeeding initiation remained at only 60% in 1995, with only 22% of mothers still breastfeeding at 6 months of age in 1995 [53]. As recently as 2004, the United States had lower breastfeeding initiation and duration rates than many continental European countries [54]. Since that time, breastfeeding in the US has increased so that in 2017, 84% of infants were ever breastfed and 58% were still breastfed at 6 months [55]. In the state of Ohio (where Cincinnati is located), in 2017 (the year that the Cincinnati PREVAIL cohort began enrollment), 80% of infants were ever breastfed and 51% were still breastfed at 6 months of age [55]. For the state of California (where Davis is located), in 2009 (the year the UC Davis Lactation Cohort began enrollment), 85% of infants were ever breastfed and 53% were still breastfed at 6 months of age [56]. Because the Davis Lactation Cohort enrolled based on the intention to breastfeed, this cohort has a longer median duration of breastfeeding than was observed in the general population (Table 2). The Cincinnati cohort had a lower duration of breastfeeding (Table 2) than that reported for Ohio, in part because this cohort is an urban, diverse cohort, which tends to mean a shorter duration of breastfeeding.

### 3.2. Bifidobacterium Species across Cohorts and Breastfeeding Patterns

**Figure 1:**
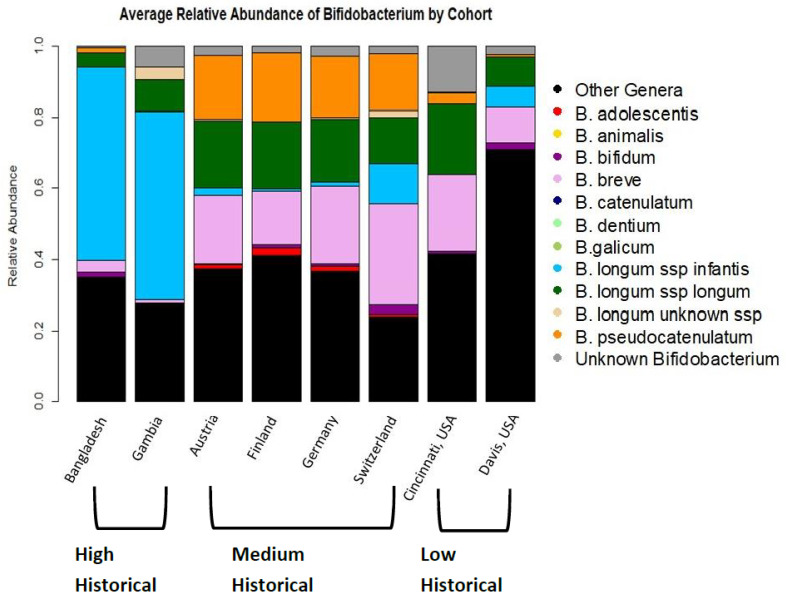
Average gut microbiome colonization patterns by country.

Average *Bifidobacterium* levels differed between cohorts (Figure 1). The prevalence of detection of any *Bifidobacterium* was 100% of infants in all cohorts except those in the United States. The total relative abundance of *Bifidobacterium* in individual infants also differed significantly by cohort (Kruskal–Wallis test, *p* < 0.0001) and by breastfeeding pattern (Kruskal–Wallis test, *p* < 0.0001). By cohort, Davis had a significantly lower total relative abundance of *Bifidobacterium* than all other cohorts (Dunn’s test, *p* < 0.0001 compared to all cohorts except Cincinnati where *p* = 0.03). Switzerland had a significantly higher relative abundance of *Bifidobacterium* than all other cohorts except for Gambia (Dunn’s test, *p* < 0.0001 for all cohorts except Gambia, no significant difference in total *Bifidobacterium* between Switzerland and Gambia, *p* = 1.0). There were no other significant differences in total *Bifidobacterium* relative abundance by cohort. By historical breastfeeding status, the low-breastfeeding-pattern infants had a lower relative abundance of *Bifidobacterium* than the high-breastfeeding-pattern infants (Dunn’s test, *p* < 0.0001) and the medium-breastfeeding-pattern infants (Dunn’s test, *p* < 0.0001). The medium-breastfeeding-pattern infants had higher total *Bifidobacterium* than the high-breastfeeding infants (Dunn’s test, *p* = 0.02).

Importantly, the cohorts differed in the species of *Bifidobacterium* that colonized infants’ guts. The average infant gut microbiome in Bangladesh and Gambia is dominated (meaning greater than 50% relative abundance *Bifidobacterium* of a single subspecies) by *B. longum* subsp. *infantis,* with an average relative abundance of 54% *B. longum* subsp. *infantis* in Bangladesh and 53% in the Gambia. In the European and USA cohorts, the average infant gut microbiome was not dominated by any single species of *Bifidobacterium* (Figure 1). 

The presence or absence of particular *Bifidobacterium* species in infants reflected historical breastfeeding practices among the cohorts (see Table 3 for the summary of findings from GEE models). Notably, *B. longum* subsp. *infantis* and *B. bifidum* were significantly less likely to be present in infants from medium- and low-breastfeeding-pattern cohorts compared to infants in high-breastfeeding-pattern cohorts. *B. pseudocatenulatum* was more likely to be present in infants from medium- or low-breastfeeding-pattern cohorts than in infants from high-breastfeeding-pattern cohorts. *B. adolescentis* and *B. breve* were detected more often in medium-breastfeeding-pattern cohorts than in high-breastfeeding-pattern cohorts, but there was no significant difference in the presence of these species between low-breastfeeding-pattern cohorts and high-breastfeeding-pattern cohorts. The prevalence of these species in each cohort is shown in Table 2. Because *B. breve*, *B. pseudocatenulatum,* and *B. bifidum* may be difficult to distinguish when using Bif-TRFLP, the values in Table 2 may underestimate the true prevalence of these taxa in all cohorts. All figures include the relative abundance of these mixed peaks as “Unknown *Bifidobacterium*”, but indistinguishable peaks do not count towards the estimates of relative abundance for these species or towards the presence or absence of these species in any infant.

**Figure 2:**
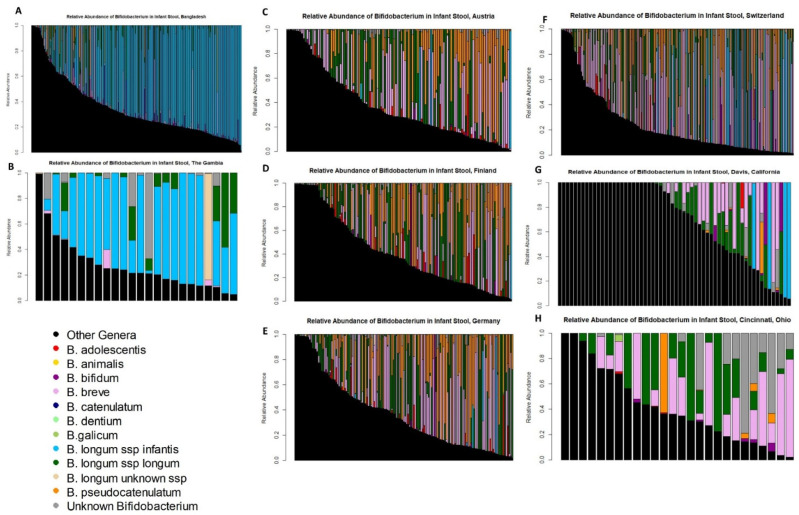
Relative abundance of different *Bifidobacterium* species in infant stools. Each bar represents the microbiome from an individual infant; cohorts with smaller numbers of infants may fail to identify the presence of rarer *Bifidobacterium* species in those cohorts by chance alone. *B. longum* subsp. *infantis* is in blue, non-*Bifidobacterium* taxa are black. (**A**) Bangladesh, (**B**) the Gambia, (**C**) Austria, (**D**) Finland, (**E**) Germany, (**F**) Switzerland, (**G**) Davis, CA, USA, (**H**) Cincinnati, OH, USA.

Detailed visualizations of *Bifidobacterium* colonization of individual infants in each cohort demonstrate the substantial concordance between historical breastfeeding patterns and infant gut colonization (Figure 2). The similarities of infant *Bifidobacterium* colonization patterns by historical breastfeeding are striking. The majority of infants in the high historical breastfeeding cohorts have gut microbiomes dominated by *B. longum* subsp. *infantis* with a lower relative abundance of other *Bifidobacterium* species (Figure 2A,B). In the medium historical breastfeeding cohorts, infants still have very high levels of total *Bifidobacterium*, but no single species dominates (Figure 2C–F). In the low historical breastfeeding cohorts, some breastfed infants completely lack *Bifidobacterium,* but those who are colonized by *Bifidobacterium* generally appear similar to the pattern observed in the medium historical breastfeeding cohorts. As medium-breastfeeding-pattern infants had higher total *Bifidobacterium* levels than high-breastfeeding-pattern infants and because there were significant differences in the presence and absence of specific *Bifidobacterium* species, we examined which *Bifidobacterium* may dominate the infant gut microbiome. In the high-historical-breastfeeding-pattern cohorts, approximately two-thirds of infants had a gut microbiome dominated (meaning greater than 50% relative abundance from a single source) by *B. longum* subsp. *infantis*. In addition, *B. breve, B. longum* subsp. *longum,* and *B. pseudocatenulatum* or a mixture of multiple *Bifidobacterium* species would occasionally result in an infant gut microbiome dominated by *Bifidobacterium.* In the medium-historical-breastfeeding-pattern cohorts, a microbiome dominated by *B. longum* subsp. *infantis* was rare, but roughly two-thirds of infants had a gut microbiome dominated by *B. breve*, *B. longum* subsp. *longum*, *B. pseudocatenulatum*, or a mixture of multiple *Bifidobacterium* species. In the low-historical-breastfeeding-pattern cohorts, some breastfed infants completely lacked any detectable level of *Bifidobacterium*. When infants did have gut microbiomes dominated by *Bifidobacterium*, it was typically the same mix of species as observed in the medium-historical-breastfeeding-pattern cohorts. On occasion, *B. bifidum* could also dominate an infant gut microbiome, but this was a rare occurrence with only one infant in the German cohort, one infant in the Finnish cohort, and one infant in the Davis, USA cohort.

### 3.3. Timing of Bifidobacterium Species Colonization

Six cohorts, namely the Bangladesh cohort, the four PASTURE cohorts (from Austria, Finland, Germany, and Switzerland), and the UC Davis lactation cohort, had additional longitudinal data on infant *Bifidobacterium* colonization patterns, although the timing of the available data differed. We analyzed these additional time points to address questions of timing regarding the acquisition of *Bifidobacterium* species (in the UC Davis cohort) and the loss or consistency of colonization with *Bifidobacterium* species comparing early infancy to 1 or 2 years of age (Bangladesh cohort and PASTURE cohorts).

**Figure 3:**
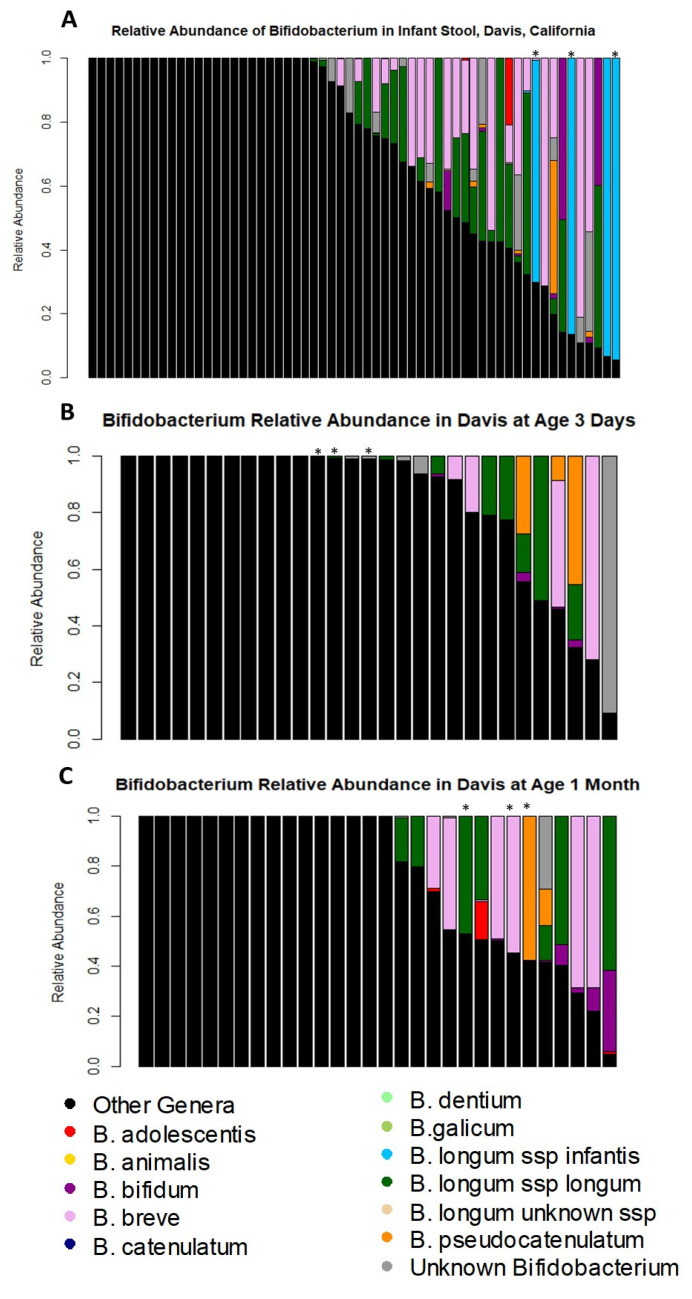
*Bifidobacterium* colonization in Davis, CA infants over time. Each bar represents the microbiome of a single infant. Stars indicate samples from infants who were colonized by *B. longum* subsp. *infantis* at age 2 months with longitudinal samples. (**A**) 2 months of age samples (repeated from Figure 2); (**B**) 3 days of age; (**C**) 1 month of age.

There were differences over time in the colonization of Davis infants with *B. longum* subsp. *infantis* (Figure 3). Figure 3A shows the 2-month *Bifidobacterium* colonization of the Davis infants, where the stars indicate the infants colonized by *B. longum* subsp. *infantis* who also had samples at an earlier time point. Regarding the timing of acquisition of *Bifidobacterium* species, the UC Davis lactation cohort had additional samples with known *Bifidobacterium* colonization patterns from day 3 of life (29 infants; Figure 3B) and month 1 of life (30 infants; Figure 3C) in addition to the month 2 samples described above. The median relative abundance of total *Bifidobacterium* was only 1% at day 3 (range 0–91%), with 38% of infants having no detectable level of *Bifidobacterium* present. The species of *Bifidobacterium* detected at day 3 of life were *B. longum* subsp. *longum* (detected in 28% of infants), *B. bifidum* (in 14% of infants), *B. breve* (in 14% of infants), *B. pseudocatenulatum* (in 14% of infants), and *B. adolescentis* (in 3% of infants) (Figure 3B). At the age of one month, 30 of the infants (including all 29 with day 3 of life samples) had additional Bif-TRFLP/BLIR results (Figure 3C). Forty-three percent of Davis infants had no detectable *Bifidobacterium* at month 1 of life, and a median relative abundance of total *Bifidobacterium* of 0.2% (range 0–95%). The species that were present were *B. longum* subsp. *longum* (detected in 23% of infants), *B. bifidum* (in 23% of infants), *B. breve* (in 23% of infants), *B. adolescentis* (in 10% of infants), and *B. pseudocatenulatum* (in 7% of infants). Notably, *B. longum* subsp. *infantis* was not found in any Davis infants prior to 2 months of age.

**Figure 4:**
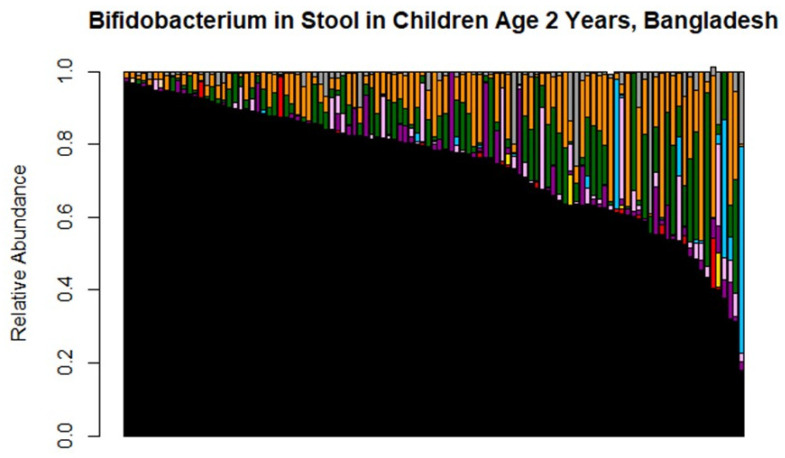
Bifidobacterium colonization in Bangladeshi cohort in infants aged 2 years. Each bar represents the microbiome of an individual infant. Slight deviations from 100% are related to rounding errors in the Bif-TRFLP calculation of percentages. Note the reduced prevalence of *B. longum* subspecies *infantis* in blue; this is a subset of the same infants shown in Figure 2B.

One hundred and nine of the 274 Bangladeshi infants had data on *Bifidobacterium* colonization at 2 years of age. At age 2, all of these infants were still colonized with at least some *Bifidobacterium*, but the median total relative abundance of *Bifidobacterium* was lower than in early life at 21% relative abundance of *Bifidobacterium* (range 3–82%; Figure 4). Furthermore, the prevalence of *B. longum* subsp. *infantis* had dropped considerably and was found in just 17% of infants overall, including 20% of still-breastfed infants and 11% of infants who had not received breastmilk in the past week (Table 4). In contrast to the dominance of *B. longum* subsp. *infantis* in early infancy, the species of *Bifidobacterium* found in the Bangladeshi infants aged 2 years, in order of decreasing prevalence, were *B. pseudocatenulatum* (detected in 95% of infants), *B. longum* subsp. *longum* (detected in 95% of infants), *B. bifidum* (detected in 78% of infants), *B. breve* (detected in 65% of infants), *B. adolescentis* (detected in 32% of infants), *B. longum* subsp. *infantis* (detected in 17% of infants), *B. animalis* (detected in 9% of infants), *B. magnum* (detected in 2% of infants), and an indistinguishable mix of *B. choerinum* and/or *B. pseudolongum* (1% of infants, and this indistinguishable mix was also plotted as part “Unknown *Bifidobacterium*”).

**Figure 5:**
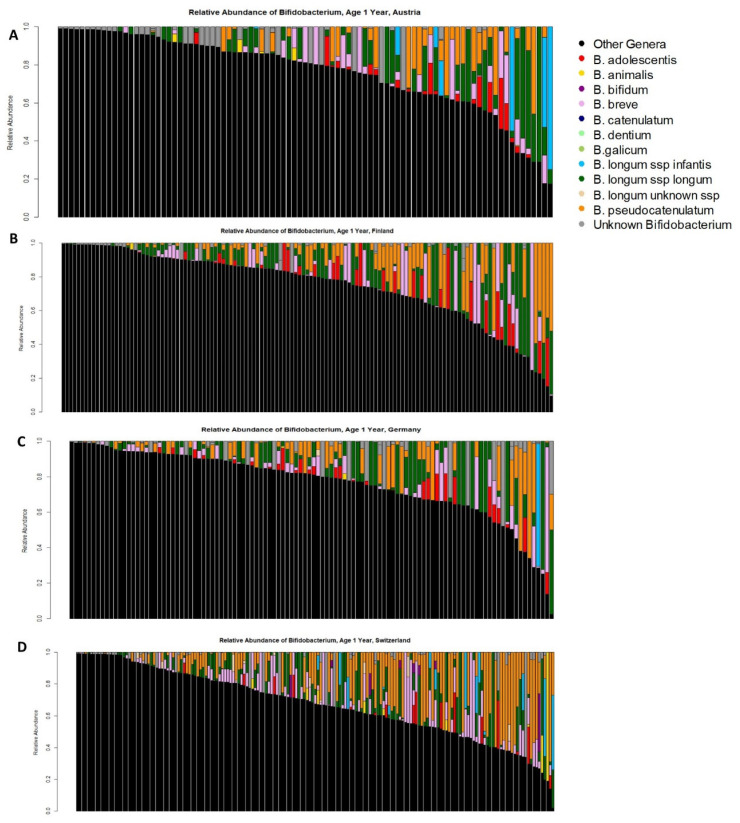
*Bifidobacterium* colonization in infants aged 1 year in (**A**) Austria, (**B**) Finland, (**C**) Germany, and (**D**) Switzerland.

In the Austrian cohort, 91 of the infants breastfed at 2 months also had a 1-year sample. In the Finnish cohort, 129 of the infants breastfed at 2 months also had a 1-year sample. In the German cohort, 110 of the infants breastfed at 2 months also had a 1-year sample. In the Swiss cohort, 175 of the infants breastfed at 2 months also had a 1-year sample. All infants were still colonized with at least some *Bifidobacterium* (Figure 5), but usually at a lower relative abundance of total *Bifidobacterium* than was present at 2 months of age. Comparing colonization with *B. longum* subsp. *infantis* between infants who were or were not still breastfed at 1 year, infants who were breastfed always had a higher prevalence of *B. longum* subsp. *infantis* than those who were not, but this difference only reached statistical significance in the Austrian (Chi-square test, *p* = 0.003) and Swiss (Chi-square-test, *p* < 0.001) cohorts (Table 4). The greater prevalence of *B. longum* subsp. *infantis* in infants breastfed at 1 year compared to infants not breastfed at 1 year in the Austrian and Swiss cohorts is consistent with horizontal transmission of *B. longum* subsp. *infantis*, as this species does not remain at high rates in infants who are no longer breastfed. Furthermore, across all the European cohorts, there were 28 infants with detectable levels of *B. longum* subsp. *infantis* at 2 months but not at 1 year, 18 infants with detectable levels of *B. longum* subsp. *infantis* at 1 year but not at 2 months, and 9 infants with detectable levels at both time points. Of the 18 infants who acquired *B. longum* subsp. *infantis* between 2 months and 1 year, 13 (72%) were breastfed at 1 year of age while only 5 (28%) were not breastfed at 1 year of age. In contrast, of the 28 infants who lost *B. longum* subsp. *infantis* between 2 months and 1 year, 26 (93%) were not breastfed at 1 year and only 2 (7%) were breastfed at 1 year. This is consistent with the idea that infants who are breastfed are more likely to acquire *B. longum* subsp. *infantis* and that this subspecies is likely to be lost after the end of breastfeeding. The mix of *Bifidobacterium* species found in these infants at 1 year of age was generally similar to those observed at 2 months (Table 5).

## 4. Discussion

In our study of cohorts from across the world, we found that infants from countries with a high historical breastfeeding pattern are more likely to be colonized with *B. longum* subsp. *infantis* and *B. bifidum* than infants from other cohorts. The pattern of *Bifidobacterium* colonization by cohort is consistent with the apparent loss of *B. longum* subsp. *infantis* in locations of the world with historically lower breastfeeding rates and shorter breastfeeding durations. We also found that the timing of colonization by *B. longum* subsp. *infantis* is distinct from the timing of colonization of other *Bifidobacterium* species. Most species of *Bifidobacterium* appeared early in the Davis cohort, suggesting a pattern of vertical transmission consistent with prior work [57]. *B. longum* subsp. *infantis*, however, did not appear as a part of any infant’s gut microbiome until 2 months of age, suggesting that this subspecies may not transmit vertically from the mother. The longitudinal data from the Bangladeshi cohort further supports our hypothesis as described in the introduction, as *B. longum* subsp. *infantis* is found in 84% of infants at 6 weeks of age, but only 17% of infants aged 2 years. This suggests that even in populations where *B. longum* subsp. *infantis* is commonly found in infants, this subspecies is unlikely to persist at older ages. This is consistent with horizontal transmission of *B. longum* subsp. *infantis* between infants. The results from the European cohorts also support this trend, as a number of infants who were still breastfed at 1 year of age acquired *B. longum* subsp. *infantis* despite not having detectable levels at 2 months of age, while *Bifidobacterium* species likely to be vertically transmitted from the mother remained at similar prevalence between 2 months and 1 year of age. 

When *B. longum* subsp. *infantis* is absent, several other *Bifidobacterium* can at least occasionally dominate the gut microbiome of individual infants. Three species are likely to contribute substantially to the dominance of total *Bifidobacterium* in the absence of *B. longum* subsp. *infantis:* *B. breve, B. longum* subsp. *longum*, and *B. pseudocatenulatum.* One species, *B. pseudocatenulatum,* is also more likely to colonize infants from medium- and low-breastfeeding countries. This suggests that this species may contribute more to the infant gut microbiome when species such as *B. bifidum* and *B. longum* subsp. *infantis,* who more frequently consume HMOs, are absent. Infants from countries with a medium breastfeeding pattern also had higher rates of colonization with *B. adolescentis* and *B. breve* than countries with a high breastfeeding pattern. In medium-breastfeeding cohorts, the open ecological niche created by the loss of *B. longum* subsp. *infantis* is filled by *Bifidobacterium* from multiple species rather than a single subspecies. The differences in species found may depend, in part, on population genetics, as, unlike *B. longum* subsp. *infantis,* other *Bifidobacterium* species are likely to consume only a subset of HMOs [58,59]. The infants from low-breastfeeding-pattern cohorts sometimes lack any detectable *Bifidobacterium*, but otherwise appear similar to the infants of medium-breastfeeding cohorts, suggesting that other species of *Bifidobacterium* may also be at risk of extinction in the absence of HMO (and therefore any microbiota-accessible carbohydrates) in the infant diet, or that other factors (for example, antibiotic use) have further challenged *Bifidobacterium* survival in these locations. Despite its rarity, when *B. longum* subsp. *infantis* is detected in infants from the medium- and low-breastfeeding-pattern cohorts, it typically dominates the infant gut microbiome of those infants, similar to the pattern observed in infants from high breastfeeding countries. 

The pattern of increased prevalence of *B. longum* subsp. *infantis* in cohorts with an uninterrupted history of long-duration breastfeeding is consistent with other studies. Consider a population in the US that never widely adopted formula: Old-Order Mennonites. This group is unusual in the United States because they have never widely adopted formula use, and generally avoid antibiotic use [60]. Consistent with what is discussed in the cohorts included in this study, colonization by *B. longum* subsp. *infantis* or with any *Bifidobacterium* is more prevalent in the Old-Order Mennonites, with *B. longum* subsp. *infantis* detected by qPCR in 70% of Old-Order Mennonite infants [1]. Furthermore, a search of the literature found the decline in the prevalence of *Bifidobacterium* in US breastfed infants occurred during the period of the 1970s with the lowest breastfeeding rates, although no explanation of the absence of *Bifidobacterium* could be found at the time [61]. Importantly, this change predates the increase in C-section delivery rates to above 10% of all deliveries in the United States [62] and before the widespread use of intrapartum antibiotics to prevent group B Streptococcus-associated sepsis [63], further suggesting diet, rather than other factors influencing the infant microbiome, drove the disappearance of *Bifidobacterium*. Also supporting the concept that antibiotic use did not drive the disappearance of *B. longum* subsp. *infantis*, antibiotic use is prevalent in Bangladesh, where approximately 40% of children under the age of 5 with acute respiratory infection receive antibiotics [64]. Despite the high rate of antibiotic use, our work shows that colonization with *B. longum* subsp. *infantis* remains prevalent in this region. 

This study does have limitations, including the small number of cohorts included. Importantly, the limited number of cohorts is due, in part, to only selecting cohorts of infants with species-level identification of *Bifidobacterium* completed using the same technique during a narrow infant age window. This careful limitation to a single method permits direct comparisons across cohorts that would not be possible if a broader range of time points or methods were included. The small number of included cohorts is balanced by the more than 900 breastfed infants included in this study. An additional strength of this study is the inclusion of cohorts from four different continents and a clear gradient in breastfeeding history across cohorts. Using Bif-TRFLP/BLIR also presents a challenge, where in some cases, species identification may be ambiguous. This imprecision in species identification is particularly common with *B. bifidum, B. breve,* and *B. pseudocatenulatum* as these three species produce peaks of very similar size. Most of the unknown *Bifidobacterium* reported in the plots is related to ambiguous peaks generated by some strains of *B. bifidum*, *B. breve,* and *B. pseudocatenulatum*. This suggests that abundance and prevalence estimates for these three species may be higher than what is reported here. *B. bifidum* occurred more frequently in Bangladesh than in any other cohort but was completely absent in the Gambia. As the number of infants in the Gambian cohort was small, this may represent a lower prevalence of *B. bifidum*, perhaps similar to what is seen in the European countries rather than a true absence. Another possibility is that *B. bifidum* is present in this cohort but goes undetected because of the similarity of *B. bifidum* to *B. pseudocatenulatum* and *B. breve* when using Bif-TRFLP. Because of these limitations in the data, it is difficult to draw conclusions about *B. bifidum* global colonization patterns from this work. The limitation in separating *B. bifidum, B. breve,* and *B. pseudocatenulatum* is counter-balanced by the improved ability to distinguish *B. longum* subsp. *infantis* from *B. longum* subsp. *longum,* even when both subspecies are found in the same infant with one at much lower levels than the other. As *B. longum* subsp. *infantis* has been proposed to be endangered in western countries [9], a method that could reliably and efficiently distinguish these two subspecies was critical to this work. Finally, there is a chance that subconscious bias influenced the classification in this work as the same scientists who analyzed the microbiome data completed the literature searches on the breastfeeding status of each country, after having seen the microbiome data. However, the differences between the groups both in breastfeeding history and in *Bifidobacterium* are substantial, and the key findings of this work, including the reduction in *B. longum* subsp. *infantis* and increases in *B. pseudocatenulatum,* are robust and detected even when the medium and low historical breastfeeding categories are combined.

*Bifidobacterium* species are important infant commensals, but the full health implications of colonization by different species of *Bifidobacterium* remains unclear. This is despite evidence that colonization by *Bifidobacterium* is important to health and evidence that some *Bifidobacterium* species may be more effective at supporting infant health than others. Some benefits of high levels of *Bifidobacterium* colonization do not appear to be species-dependent, such as the reduced carriage of antimicrobial resistance genes [4]. Other benefits of high levels of *Bifidobacterium* are species-dependent. For example, higher levels of *B. longum* subsp. *infantis* are associated with improved vaccine response, but the same association is not seen with higher levels of *B. longum* subsp. *longum* or *B. breve* [3]. *B. longum* subsp. *longum* is found more often in healthy infants than in those with allergic symptoms, but the same trend was not seen for other *Bifidobacterium* species [65]. *B. longum* subsp. *infantis* was found more frequently in Old-Order Mennonite infants with lower risk of atopic disease than in infants from a nearby Rochester (Rochester, NY, USA) cohort [1]. As such, understanding the differences in *Bifidobacterium* species colonization in infant populations is important when studying the health implications of the early life microbiome. The fact that the health implications of *Bifidobacterium* vary by species and that *Bifidobacterium* species colonization patterns vary by country and population history of breastfeeding means that care needs to be taken in interpreting health findings associated with high levels of *Bifidobacterium* without also identifying species if trying to apply the findings to other populations. This means it is critical to understand the epidemiological factors that support the transmission of different species of *Bifidobacterium*, including considering how historic formula-driven extinctions may have changed the colonization patterns observed in the present day.

## Figures and Tables

**Table 1:** Source of included cohorts, and number of infants included in the present study from each cohort.

Country of Origin	Published	Study	Enrollment Based on Intent to Breastfeed?	Total Number Infants in Cohort	Number of Infants with Stool Samples	Number of Breastfed Infants at Time of Sample Collection
Austria	Partially	PASTURE	No	207	181	122
Bangladesh	Yes	Efficacy of Newborn Vitamin A Supplementation in Improving Immune Function (clinicaltrials.gov NTC01583972)	No	306	274	274
Finland	Partially	PASTURE	No	171	153	135
Gambia	Yes	Sub-study in The Early Nutrition and Immune Development (ENID) Trial, ISRCTN49285450	Yes	33	24	24
Germany	Partially	PASTURE	No	237	198	149
Switzerland	Partially	PASTURE	No	231	227	189
Davis, CA, USA	Partially	UC Davis Lactation Cohort	Yes	95	60	60
Cincinnati, OH, USA	No	PREVAIL	No	245	45	26

**Table 2:** Summary of *Bifidobacterium* prevalence in infants aged 1 to 2 months. Except for information on breastfeeding initiation and duration in a cohort, values are based solely on infants who were breastfed. The breastfeeding initiation (ever breastfed) rate and breastfeeding duration were calculated using data from all infants in a cohort with an available sample and a known breastfeeding status.

Cohort Location	Ever Breastfed (Median Duration)	Historic Breastfeeding Pattern	Any *Bifidobacterium*	*B. adolescentis*	*B. animalis*	*B. bifidum*	*B. breve*	*B. longum* subsp. *infantis*	*B. longum* subsp. *longum*	*B. longum* subsp. Unknown	*B. pseudocatenulatum*
Bangladesh	100% (>2 years)	High	100%	5.8%	2.6%	32.5%	40.1%	83.6%	26.3%	1.1%	8.4%
Gambia	100% (Unknown)	High	100%	0%	0%	0%	25%	91.7%	54.2%	12.5%	0%
Austria	91.8% (6.8 months)	Medium	100%	23.7%	0.82%	6.6%	55.7%	4.1%	61.5%	4.9%	52.4%
Finland	99.3% (8 months)	Medium	100%	15.5%	0%	8.8%	40.0%	0.74%	56.3%	3.7%	51.1%
Germany	91.1% (7.4 months)	Medium	100%	18.1%	0.67%	7.4%	61.1%	4.0%	61.7%	4.0%	57.0%
Switzerland	97.3% (8 months)	Medium	100%	4.8%	0%	11.1%	58.7%	14.8%	41.8%	6.3%	49.7%
Davis, CA, USA	100% (9.3 months)	Low	65%	8.3%	0%	13.3%	36.7%	8.3%	36.7%	1.7%	15%
Cincinnati, OH, USA	86.5% (3.1 months)	Low	97%	11.5%	0%	19.2%	61.5%	0%	69.2%	0%	19.2%

**Table 3:** Summary of GEE models comparing presence or absence of *Bifidobacterium* species in individual infants by cohort history of breastfeeding. Reference group was the high-breastfeeding group. To account for multiple comparisons, a *p*-value less than 0.0062 is considered significant. The model for *B. animalis* failed to run, because after excluding infants who were not breastfed at time of sample collection, there were no infants from a low historical breastfeeding pattern cohort colonized by *B. animalis.* Taxa that differed significantly in prevalence from the high historical breastfeeding pattern cohorts are in bold.

Cohort Breast-Feeding Pattern	*B. adolescentis* Odds Ratio (95% CI, *p*-Value)	*B. animalis* Odds Ratio (95% CI, *p*-Value)	*B. bifidum* Odds Ratio (95% CI, *p*-Value)	*B. breve* Odds Ratio (95% CI, *p*-Value)	*B. longum* Subspecies *infantis* Odds Ratio (95% CI, *p*-Value)	*B. longum* Subspecies *longum* Odds Ratio (95% CI, *p*-Value)	*B. longum* Unknown Subspecies Odds Ratio (95% CI, *p*-Value)	*B. pseudocatenulatum* Odds Ratio (95% CI, *p*-Value)
Medium	**4.1 (1.6–11, *p* = 0.0041)**	NA	**0.22 (0.16–0.31, *p* < 0.0001)**	**2.0 (1.3–3.1, *p* = 0.0019)**	**0.010 (0.0037–0.029, *p* < 0.0001)**	2.2 (0.98–5.0, *p* = 0.055)	2.8 (0.92–8.2, *p* = 0.069)	**12 (10–13, *p* < 0.0001)**
Low	2.4 (1.0–5.6, *p* = 0.041)	NA	**0.40 (0.28–0.58, *p* < 0.0001)**	1.4 (0.70–2.9, *p* = 0.32)	**0.0084 (0.0024–0.029, *p* < 0.0001)**	1.8 (0.57–5.7, *p* = 0.31)	0.63 (0.16–2.4, *p* = 0.51)	**2.1 (1.8–2.5, *p* < 0.0001)**

**Table 4:** Table of number of European infants colonized at any level by *B. longum* subsp. *infantis* at 1 year of age by breastfeeding status. Prevalence of *B. longum* subsp. *infantis* appears to decrease rapidly during the weaning period, supporting the assumption that vertical transmission will be rare. *p*-values are for the Chi-square tests comparing *B. longum* subsp. *infantis* detection in still breastfed vs. no longer breastfed infants.

Country	Breast Fed at 1 Year (European Countries) or 2 Years (Bangladesh)	*B. longum* Subspecies *infantis* Detected	*B. longum* Subspecies *infantis* Not Detected	Percentage of Infants Colonized
Austria (*p* = 0.003)	Yes	5	14	36%
	No	2	70	2.8%
Finland (*p* = 0.65)	Yes	1	37	2.6%
	No	0	91	0%
Germany (*p* = 1)	Yes	1	18	5.3%
	No	2	89	2.2%
Switzerland (*p* < 0.0001)	Yes	10	24	29%
	No	6	135	4.3%
Bangladesh (*p* = 0.35)	Yes	13	51	20.3%
	No	5	40	11.1%

**Table 5:** Summary of *Bifidobacterium* prevalence in infants aged 1 year.

Cohort Location	Any *Bifidobacterium*	*B. adolescentis*	*B. animalis*	*B. bifidum*	*B. breve*	*B. longum* subsp. *infantis*	*B. longum* subsp. *longum*	*B. longum* Unknown Subspecies	*B. pseudocatenulatum*
Austria	100%	23%	5.5%	1.1%	35%	7.7%	53%	0%	36%
Finland	100%	25%	2.3%	0%	38%	0.8%	70%	0%	47%
Germany	100%	27%	3.6%	0%	44%	2.7%	67%	0.9%	52%
Switzerland	100%	9.1%	6.8%	4.6%	51%	9.1%	62%	7.4%	61%

## Data Availability

Bangladesh cohort sequencing data can be found in SRA under accession IDs PRJNA636907 and PRJNA636905. The Gambia cohort sequencing data can be found in QIITA under study ID 10297 and the European Nucleotide Archive under accession number ERP017462. PASTURE cohort sequences can be found as part of the supplementary data of DOI 10.1038/s41591-020-1095-x without metadata; PASTURE is a cohort with ongoing field cohort. As long as the cohort is ongoing, European data protection prohibits sharing of individual data, even if pseudonymized. The Davis Lactation cohort data can be found in SRA under accession ID PRJNA820048. The PREVAIL (Cincinnati) cohort data can be found in SRA under accession ID PRJNA819967.
